# The Renin-Angiotensin-Aldosterone System in Vascular Inflammation and Remodeling

**DOI:** 10.1155/2014/689360

**Published:** 2014-04-06

**Authors:** Maricica Pacurari, Ramzi Kafoury, Paul B. Tchounwou, Kenneth Ndebele

**Affiliations:** ^1^Biology Department, College of Engineering, Science, and Technology, Jackson State University, Jackson, MS 39217, USA; ^2^NIH RCMI-Center for Environmental Health, College of Engineering, Science, and Technology, Jackson State University, Jackson, MS 39217, USA

## Abstract

The RAAS through its physiological effectors plays a key role in promoting and maintaining inflammation. Inflammation is an important mechanism in the development and progression of CVD such as hypertension and atherosclerosis. In addition to its main role in regulating blood pressure and its role in hypertension, RAAS has proinflammatory and profibrotic effects at cellular and molecular levels. Blocking RAAS provides beneficial effects for the treatment of cardiovascular and renal diseases. Evidence shows that inhibition of RAAS positively influences vascular remodeling thus improving CVD outcomes. The beneficial vascular effects of RAAS inhibition are likely due to decreasing vascular inflammation, oxidative stress, endothelial dysfunction, and positive effects on regeneration of endothelial progenitor cells. Inflammatory factors such as ICAM-1, VCAM-1, TNFα, IL-6, and CRP have key roles in mediating vascular inflammation and blocking RAAS negatively modulates the levels of these inflammatory molecules. Some of these inflammatory markers are clinically associated with CVD events. More studies are required to establish long-term effects of RAAS inhibition on vascular inflammation, vascular cells regeneration, and CVD clinical outcomes. This review presents important information on RAAS's role on vascular inflammation, vascular cells responses to RAAS, and inhibition of RAAS signaling in the context of vascular inflammation, vascular remodeling, and vascular inflammation-associated CVD. Nevertheless, the review also equates the need to rethink and rediscover new RAAS inhibitors.

## 1. Renin-Angiotensin-Aldosterone System (RAAS) and Cardiovascular Disease

The rennin-angiotensin-aldosterone system (RAAS), one of the most important hormonal systems, oversees the functions of cardiovascular, renal, and adrenal glands by regulating blood pressure, fluid volume, and sodium and potassium balance [1]. The classical RAAS system was discovered more than a century ago, and in 1934 Goldblatt et al. showed a Renin link between kidney function and blood pressure [2]. Since then, extensive experimental studies have been undertaken to identify the components of the RAAS and its role in regulating blood pressure. Abnormal activity of the RAAS leads to the development of an array of cardiovascular diseases (CVD; hypertension, atherosclerosis, and left ventricular hypertrophy), cardiovascular events (myocardial infarction, stroke, and congestive heart failure), and renal disease [1]. As early as in 1956, Leonald T. Skeggs suggested the development of drugs to regulate renin-angiotensin-system (RAS), and since then an array of inhibitors have been developed. Due to RAAS signaling pathways complexity than previously thought, half-century later, new RAAS inhibitors are still being developed [3]. Indeed, numerous experimental and clinical evidences indicate that pharmacological inhibition of RAAS with angiotensin-converting enzyme inhibitors (ACEIs), angiotensin receptor blockers (ARBs), direct rennin inhibitors (DRIs), and mineralocorticoid receptor antagonists (MRAs) is effective in treating hypertension and diabetic renal injury, and the results show a reduction in CVD and heart-related events worldwide [1]. This review discusses recent findings in our understanding of the role of RAAS components and their inhibition effects on vascular inflammation, vascular remodeling, and CVD.

### 1.1. RAAS

Renin, an active proteolytic enzyme, is first synthesized as an inactive preprohormone (prorenin), undergoes subsequent proteolytic changes in the afferent arterioles of renal glomerulus, and then is released into circulation [4]. In the circulation, proteolytic and nonproteolytic mechanisms cleave prorenin to the active renin. Active renin acts upon its substrate, angiotensinogen, to generate angiotensin I (Ang I). Ang I is cleaved by angiotensin-converting enzyme (ACE) resulting in physiologically active angiotensin II (Ang II). Ang II, the main effector of the RAAS, mediates its effects via type 1 Ang II receptor (AT1R). However, few studies suggest the existence of additional receptors for prorenin and renin in the heart, kidney, liver, and placenta [5]. Other studies suggest the presence of renin receptors in visceral and subcutaneous adipose tissues suggesting a local production of Ang II. Activation of prorenin and renin receptors stimulates mitogen activated kinase (MAPK)/extracellular signal-regulated kinase (ERK1/2) related signaling pathway [6]. Since the rate-limiting step of RAAS is under the control of renin, the idea of inhibiting renin to suppress RAAS was suggested in the mid-1950s, but the development of rennin inhibitors was a long and difficult process [7]. Likewise, the first oral DRI, aliskiren, was marketed in 2007 for the treatment of hypertension [8]. Another effector of the RAAS, aldosterone, exerts important endocrine functions by regulating fluid volume, sodium and potassium homeostasis, and primarily acting in the renal distal convoluted tubules. Aldosterone mediates genomic and nongenomic effects via mineralocorticoid receptor (MR), AT1R, G-protein-coupled receptor, and epidermal growth factor receptors (EGFR). Downstream effectors of these receptors such as MAPK/ERK1/2/p38 pathways mediate vascular biology and physiology, particularly, vascular remodeling, inflammation, fibrosis, and vascular tone. Aldosterone's cardiopathological effects include myocardial fibrosis and hypertrophy and vascular remodeling and fibrosis. Production of aldosterone is under the regulation of angiotensin II, hyperkalemia, adrenocorticotropic hormone (ACTH), and sodium level [9]. Clinical trials have shown that blocking aldosterone receptors with mineralocorticoid receptor antagonists (MRA), spironolactone or eplerenone, reduces blood pressure, lowers albuminuria, and improves the outcome of patients with heart failure or myocardial infarctions or cardiovascular complications associated with diabetes mellitus [10]. Aldosterone infusion in an ischemia animal model induces vascular changes via AT1R, since blocking AT1R inhibited aldosterone effects, indicating cross-talk among RAAS components.

The recent discovery and cloning of a new angiotensin converting enzyme, ACE2, has introduced further complexity to RAAS. ACE2 is 42% homolog to ACE1 and is expressed in the heart, kidney, testis, endothelium of coronary, intrarenal vessels, and renal tubular epithelium [11]. ACE2 is a monopeptidase with enzymatic preference for hydrophobic/basic residues of Ang II C-terminus that leads to the formation of angiotensin II (1–7). Experimental studies show that Ang II (1–7) is a competitor of Ang II and indeed may have cardiorenal protective effects [12, 13]. Ang II is also produced by non-ACE enzymes, such as serine protease chymase, which have been found in the heart, vasculature, and other tissues [14, 15].

## 2. Inflammation and Cardiovascular Disease

Inflammation plays a key role in the initiation, progression, and development of an array of cardiovascular diseases such as hypertension, atherosclerosis, restenosis after balloon angioplasty, nephropathy, and cardiomyopathy [16]. A typical example of how inflammation underlies the development of cardiovascular disease is atherosclerosis, via the activation of endothelial cells by the inflammatory cytokines. Endothelium dysfunction due to injury by the inflammatory process has been associated with cardiovascular risk factors including hypertension, diabetes mellitus, or obesity [17].

### 2.1. Markers of Inflammation

Tumor necrosis factor alpha (TNF*α*) is a key proinflammatory cytokine regulating the expression of many genes of inflammation, oxidative stress, and antiapoptotic signaling pathways, virtually, in all types of cells [18]. Aberrant TNF*α* signaling leads to the development of pathological conditions including cardiovascular disease, and therapeutic blocking of TNF*α* signaling has been proposed for the treatment of several inflammatory diseases particularly rheumatoid arthritis and bowel disease [18]. TNF*α* impairs endothelium-dependent nitric oxide (NO) mediated vasorelaxation in coronary arteries or carotid artery via superoxide radical production [19]. Patients with high levels of circulating TNF*α* have a greater risk of developing cardiovascular disease [20]. In endothelial cells, TNF*α* induces the expression of interleukin-6 (IL-6), monocyte chemoattractant protein-1 (MCP-1), and cell adhesion molecules (CAM) [21]. Deletion of TNF*α* in mice inhibits intimal hyperplasia after carotid artery injury [22], while an increased expression of TNF*α* aggravates pulmonary hypertension in mice [23]. TNF*α* mediated inflammation plays an important role in vascular remodeling. Human carotid artery smooth muscle cells respond to TNF*α* with increased cell proliferation, whereas inhibition of circulating TNF*α* prevents carotid artery postinjury media remodeling and neointima formation in rats [24]. TNF*α* inhibition has been shown to improve endothelium function via stimulating endothelial cells regeneration [25].

NF-*κ*B, a proinflammatory factor downstream of TNF*α*, plays a central role in regulating the expression of vascular inflammatory mediators interleukin-1 beta (IL-1*β*), interleukin-6 (IL-6), TNF*α*, and MCP-1 in endothelial cells and other cell types [26]. Activated NF-*κ*B induces vascular smooth muscle cells proliferation and mediates neointima hyperplasia after vascular injury [27].

Another marker of inflammation is C-reactive protein (CRP). CRP is considered a hallmark of the acute-phase response and a predictor of cardiovascular event risk [28]. C-reactive protein is mainly produced in the liver [29], but other cell types such as smooth muscle and endothelial cells of atherosclerotic arteries show CRP expression [30]. CRP plays a role in mediating vascular disease.* In vitro* studies show that CRP has proinflammatory and prothrombotic effects [31], inhibits endothelial progenitor cell differentiation and function [32], and upregulates AT1R [33]. CRP activates classical complement signaling cascade, which plays a key role in neointima formation in injured vessels [34]. Circulating CRP levels correlate with several inflammation markers including inflammatory cytokines, cell adhesion molecules, markers of activated platelets, and white cells [35]. All of these inflammation markers are also predictive of coronary artery events [36].

Interleukin-6, a pleiotropic cytokine, regulates many cellular functions including proliferation and apoptosis. IL-6 plays an important role in inflammation and modulates the development of several diseases including cardiovascular disease such as hypertension and other related diseases. High circulating levels of IL-6 are found in hypertensive patients. Type I diabetic rats have high circulating levels of IL-6 and increased blood vessel contractility [37]. IL-6 overexpression in mice induces pulmonary vascular remodeling that is similar to that seen in patients with pulmonary hypertension and induces pulmonary hypertension via proliferative and antiapoptotic mechanisms [38]. IL-6 also modulates vascular reactivity. Treatment of isolated human blood vessels from various organs with IL-6 results in increased contraction [39]. IL-6 mediates the development of vascular occlusive disease and is a predictor of cardiovascular sudden death [40]. IL-6 effects on vascular system are mediated via NF-*κ*B signaling, which plays a key role in vascular remodeling. Inhibition of NF-*κ*B via deletion of I*κ*BNS, a nuclear I*κ*B regulatory protein of NF-*κ*B, reduces intimal hyperplasia after vascular injury in mice via NF-*κ*B-mediated IL-6 production [41].

### 2.2. Intercellular Adhesion Molecules

Inflammation-mediated injury to endothelium generates a pronflammatory signaling cascade and the expression of intercellular adhesion molecule-1 (ICAM-1) and vascular cell adhesion molecule-1 (VCAM-1), both of which recruit blood monocytes to vascular wall, thus perpetuating the release of more cytokines and chemokines at injury site culminating with development of vascular disease such as atherosclerosis. Circulating levels of ICAM-1 and VCAM-1 positively correlate with carotid intima-media ratio [42]. VCAM-1 expression is upregulated by Ang II in rat aorta, whereas spironolactone, an antagonist of mineralocorticoid receptors, inhibits VCAM-1 and other inflammatory markers expression [43]. Treatment of endothelial cells with Ang II upregulates VCAM-1 via oxidative stress and NF-*κ*B activation [44]. High circulating levels of ICAM-1, VCAM-1, and other inflammatory cell adhesion molecules are associated with left ventricle hypertrophy (LVH) and diastolic dysfunction in aged population [45].

## 3. RAAS and Vascular Inflammation

RAAS plays a crucial role in the initiation and maintenance of vascular inflammation and vascular remodeling. Vascular inflammation leads to endothelium dysfunction, and a decreased endothelial function mediates progression of cardiovascular disease. A dysfunctional endothelium is leaky and facilitates migration of inflammatory cell into the vascular wall and stimulates smooth muscle cells proliferation, processes that decrease vascular function and promote development of cardiovascular disease and tissue injury. A dysfunctional endothelium provides proinflammatory environment in such as it promotes recruitment and attachment of inflammatory cells, which are well known to play a key role in atherosclerosis. There is increasing evidence indicating a link between hypertension and atherosclerosis via Ang II mediated inflammation.* In vivo*, acute treatment with Ang II significantly increases leukocytes adhesion in the rat mesenteric arteries [46]. Animal and human studies show that Ang II has proinflammatory responses in arteries, heart, and kidney by regulating the expression of cytokines and chemokines. In human vascular smooth muscle cells, Ang II induces NF-*κ*B activation and the expression of IL-6 [47], MCP-1, and TNF*α* in monocytes [48].* In vivo* infusion of Ang II causes increased expression of VCAM-1 in rat aorta via NF-*κ*B transcriptional activation. Administration of losartan, an AT1R antagonist, inhibits Ang II-induced NF-*κ*B activation and VCAM-1 accumulation [49].* In vitro* treatment of rat vascular smooth muscle cells with Ang II upregulates MCP-1, and blockade of AT1R with losartan prevents MCP-1 expression and monocytes migration into vessel wall and other target organs [50]. Although a vasoconstrictor, Ang II induces endothelial damage by inhibiting endothelial cells regeneration. Ang II acts as a second messenger to activate intracellular signaling pathways such as mitogen-activated protein kinase (MAPK) and AKT, pathways that mediate cell proliferation and apoptosis and thereby vascular dysfunction [51]. Ang II plays a significant role in the initiation and progression of atherogenesis, an inflammation mediated process. In injured arteries, Ang II provides a positive feedback loop in vascular inflammation via recruitment of inflammatory cells, which then produce more Ang II, therefore perpetuating vascular inflammation [1]. Ang II is a potent prooxidant. Ang II induces the production of superoxide anions and activates the prooxidant NADH/NADPH signaling [52]. Ang II-mediated oxidative stress reduces nitric oxide (NO) level and activates redox sensitive genes, particularly cytokines, adhesion molecules, and matrix metalloproteinases [53]. Ang II is also a profibrotic factor. Chronic infusion of mice with Ang II results in increased blood pressure, infiltration of inflammatory cells into myocardium, and cardiac fibrosis [54]. In rat cardiomyocytes, Ang II induces calcium signals (Ca^2+^) and oxidative stress, which cooperatively induce cardiomyocytes hypertrophy [55]. Chronic treatment of rat aortic smooth muscle cells with Ang II induces cell hypertrophy by increasing protein synthesis [56]. Ang II-treated rat cardiac fibroblasts display increased expression of focal adhesion kinases (FAK) and integrins, whereas cardiac myocytes express high levels of c-fos, EGFR1, TGF*β*, and extracellular matrix proteins (109, 110). Inflammation mediates endothelial injury which alters endothelial cell architecture so that the endothelium becomes dysfunctional. It has been shown that a dysfunctional endothelium is directly associated with hypertension and atherosclerosis [17]. A functional endothelium is a key regulator of NO release, and loss of NO bioavailability is associated with high level of Ang II via oxidative stress. Although development of atherosclerosis is a multifactorial complex process, interaction between endothelial dysfunction and oxidative stress plays an important role in atherosclerotic process. Increased oxidative stress within the vascular wall is a hallmark of vascular disease such as atherosclerosis, hypertension, and diabetes. Indeed, high level of superoxide is an important factor in atherosclerosis initiation by recruitment of inflammatory cells and endothelial dysfunction. Total genetic deletion of NADPH oxidase subunit, Nox2, in mice results in significant decrease of aortic atherosclerosis [57]. Blocking RAAS with valsartan in combination with fluvastatin (a statin) in atherosclerosis mouse model, the apolipoprotein E (ApoE^−^/^−^) null mice, reduces the level of atherosclerotic lesions, superoxide anion, and the expression level of MCP-1 and ICAM-1, indicating that blocking inflammation and oxidative stress has beneficial effects on endothelium [58]. Indeed, clinical studies show a reduction in cardiovascular events beyond blood pressure lowering such as positively altering endothelium/vascular wall structure which in turn mediates reduction of cardiovascular disease. Several RAAS inhibitors such as ACEI ramipril and ARB losartan improve endothelial activity and vascular function by increasing NO bioavailability [17]. NO has protective effects on cardiovascular and renal systems. NO effects on the vasculature are numerous from inducing vasodilatation of all types of blood vessels to inhibiting platelet aggregation and adhesion or leukocytes adhesion to endothelium. Furthermore, NO inhibits DNA synthesis, mitogenesis, and vascular smooth muscle cells proliferation and counteracts oxidative stress [59]. NO bioavailability depends on the activity of eNOS, and a diminished eNOS activity is associated with essential hypertension [59].

**Figure 1:**
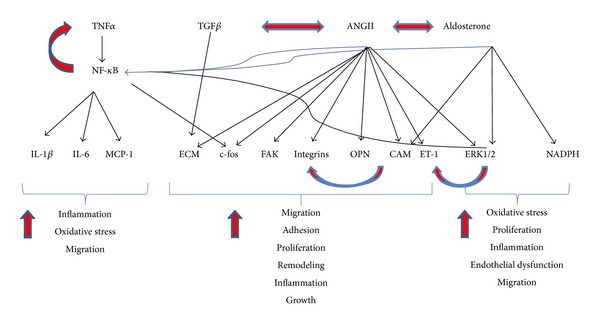
Schematic representation of various molecular factors activated by RAAS effectors and cross-talk between RAAS effectors and molecular factors involved in signaling pathways with role in vascular inflammation and remodeling. Double arrow: cross-talk; single arrow: increased expression or stimulation.

The proinflammatory and profibrotic effects of the RAAS are also mediated by aldosterone. Aldosterone plays a role in organ fibrosis and tissue ischemia, and in conjunction with macrophages, it induces cardiac fibrosis [60]. Aldosterone promotes insulin resistance and vascular remodeling and influences the development of atherosclerosis [61]. In vascular smooth muscle cells, aldosterone alters insulin signaling by upregulating the expression of insulin-like growth factor-1 receptor (IGF1R) and hybrid receptor and modulates membrane structure via tyrosine kinase receptors [62]. Chronic infusion of aldosterone induces oxidative stress in rat aorta, and MR antagonist spironolactone reduces reactive oxygen species generation [62]. Animal studies also indicate an association between aldosterone and decreased NO synthesis and endothelial progenitor cells (EPC) via oxidative stress and low levels of VEFGR2 [63]. NO plays a key role in vascular homeostasis through its effects on endothelial and smooth muscle cells. In endothelial cells, NO potentiates VEFG mitogenic effects, thereby stimulating endothelial cells proliferation. In VSMC, NO limits their proliferation and migration [64]. In addition to RAAS present in systemic circulation and its production in local tissues, there are also reports about the identification of an intracellular RAAS in certain cell types such as hepatoma cells [65], renal cortical cells [66], or adrenal medullary chromaffin and pituitary glandular cells [67]. Human and rat adrenal cortical cells stimulated with Ang II produce aldosterone via AT1R-upregulation of cytochrome P450 oxidase B2 and increased level of hydrogen peroxide, whereas pretreatment with losartan and antioxidants abrogates Ang II effects [68]. As shown in Figure 1, Ang II, the master cytokine, TNF*α*, and aldosterone induce the expression of a myriad of molecular effectors of signaling pathway associated with vascular inflammation and remodeling, fibrosis, and oxidative stress. Several molecular molecules such as ERK1/2 and NADPH are also activated by Ang II and aldosterone and activate NF-*κ*B-dependent signaling in the absence of TNF*α*, cross-talk that indicates the complexity of RAAS effectors role in mediating vascular inflammation and remodeling (Figure 1). Moreover, the cross-talk between Ang II and aldosterone indicates the intricacy of the RAAS system on cardiovascular system pathology.

## 4. RAAS Blockers and Vascular Inflammation

Blocking RAAS signaling either with ACEIs which inhibit the formation of angiotensin, or ARBs which block angiotensin receptors, or DRIs which inhibit the renin-angiotensinogen reaction, or MRAs which block aldosterone, alone or in combinations, reduces mortality and morbidity in diabetes, hypertension, atherosclerosis, heart failure, and stroke [69]. The multiple biological and physiological effects as a result of RAAS inhibitors are summarized in Table 1 and include decreased inflammation, vascular remodeling, and fibrosis, oxidative stress, increased endothelial function and nitric oxide, and maintenance of bradykinin and endothelium-derived hyperpolarizing factors (EDHF), both of which contribute to maintenance of vascular tone. However, blockade of the RAAS at one level is not very effective to treat hypertension; therefore blocking RAAS at multiple levels seems to provide clinical efficacy for the treatment of hypertension and other forms of cardiovascular disease including atherosclerosis [69].

### 4.1. Direct Renin Inhibitors (DRIs)

DRIs block RAAS by inhibiting renin enzymatic activity. A recently approved DRI, aliskiren, is an oral direct renin inhibitor that lowers blood pressure by blocking the rate-limiting step of the RAAS. In a randomized double-blinded trial study, Andersen et al. [70] and others [71] reported that aliskiren-based therapy lowered blood pressure (BP) and plasma renin activity (PRA), and the effects persisted over four weeks, suggesting long term effects of aliskiren on renin activity. However, plasma renin activity can be increased by ACEIs or ARB; therefore combination of aliskiren with ACEIs or ARBs has been considered as a preferred option of treatment of hypertension, congestive heart failure, and chronic kidney disease [72]. Aliskiren has beneficial effects on endothelium. In patients with type I diabetes, aliskiren improved endothelial function independently of lowering blood pressure. In an atherosclerosis transgenic mouse model, aliskiren alone or in combination with atorvastatin inhibited atherosclerosis development and plaque progression via decreasing monocytes adhesion and MCP-1 levels [73]. In eNOS deficient mice, aliskiren prevented cardiac hypertrophy, inflammation, coronary artery remodeling, and vascular intimal hyperplasia, and even greater effects were found in combination with valsartan. Mechanistically, aliskiren and valsartan combination downregulates NADPH oxidase activity and therefore attenuates oxidative stress [59], which plays a key role in initiating the development of vascular inflammation and cardiovascular disease.

### 4.2. (Pro)renin Receptor and Potential Inhibitors

(Pro)renin receptor (P)RR discovery in 2002 has shed light on tissue renin-angiotensin system and function of circulating prorenin, the inactive precursor of renin. Prorenin and renin have been shown to bind (P)RR with high affinity; however, prorenin exhibits higher affinity for (P)RR, an indication that indeed prorenin might be the actual ligand for (P)RR. Studies have shown that upon prorenin binding and activation of (P)RR, downstream signaling results in the activation of MAPK ERK1/2/p38 and Akt in a variety of cells, including VSMC [74]. The role of (P)RR in cardiovascular pathology proved to be more difficult to study than thought, since genetic deletion of (P)RR in mice is lethal or life-span is very short, making functional analysis of this receptor almost impossible to study and also suggesting that there is a need of normal level of (P)RR for homeostasis. However, several studies focused on (P)RR role in cardiovascular complications mediated by diabetes. Diabetic nephropathy significantly increases (P)RR level in kidneys, and use of (R)RR blocker, HRP, partially outcompeted renin and prorenin binding to (P)RR, normalized Ang II levels, or attenuated existing nephropathy [75, 76]. In hypertensive animal models, HRP improved kidney and left ventricle function and decreased fibrosis. However, HRP failed to decrease blood pressure or renal injury in more complicated animal models, particularly in animal models with overexpressed prorenin and angiotensinogen [77]. In VSMC overexpressing human (P)RR, HRP failed to block prorenin binding to (P)RR or ERK1/2 activation, whereas in human umbilical vein endothelial cells, HRP failed to bind to (P)RR [77, 78]. It is suggested that in patients with hemodialysis and increased cardiovascular death, inhibition of (P)RR in addition to ACEI or ARB might have the potential to positively reduce heart hypertrophy or protein excretion at least based on animal studies; however, HRP reversed the effects of DRI on blood pressure or coronary circulation [79]. Based on information presented above and complexity of RAS signaling or RAS inhibition negative feedback loop, HRP clinical use must first thoroughly understood* in vitro* and* in vivo* studies before clinical studies ensue.

### 4.3. Angiotensin Converting Enzyme Inhibitors (ACEIs)

The use of ACEIs is an effective conventional treatment of hypertension and reducing left ventricular hypertrophy, therefore, ACEIs improve CV outcomes [80] ACEIs treatment of patients with a dysfunctional endothelium caused by various pathological conditions improves endothelial functions measured by brachial flow mediated vasodilation (FMD) [81]. Mechanistically, ACEIs improve endothelium function by increasing NO level via blocking bradykinin degradation and inhibit the production of endothelin-1 (ET-1) and Ang II by endothelium [82]. Maintaining bradykinin level has an additive effect on endothelium by increasing the level of prostacyclin and EDHF, both of which induce vasodilation and inhibit vascular smooth muscle cells proliferation and platelet adhesion [82–84]. Most recent evidence suggests that oxidative stress, inflammation, and hypertension are interrelated processes that influence each other in the pathology of hypertension-related cardiovascular events. Therefore, new inhibitors are being developed to target ACE and particularly to have antioxidant activity; so in concert the new properties of one inhibitor may contribute to endothelium-dependent protective effects against oxidative stress and high blood pressure. Several selenium analogues of ACE inhibitor, captopril, have been developed and shown to have ACE inhibitory effect and to scavenge oxidative stress product, peroxynitrite [85, 86]. Although these new ACE inhibitors show promising inhibitory effects, extended* in vitro* and* in vivo* studies are necessary to understand and determine their extended physiological effect.

### 4.4. Ang 1–7 and Mas Axis

In addition to Ang II, other Ang peptides Ang III (Ang-2–8), Ang IV (Ang 3–8), and Ang 1–7 have biological effect within RAAS system [87]. Tissue and circulating Ang 1–7 level is similar to that of Ang II; however, unlike Ang II, Ang 1–7 is a vasodilator and mediates antiangiogenic and antimitogenic effects [87]. Ang 1–7 inhibits smooth muscles proliferation and inhibits neointima formation following carotid injury or abdominal aorta stenting [88, 89]. Ang 1–7 beneficial cardiovascular effects are mediated via G protein-coupled receptor Mas (MasR). MasR have been shown to be present in human endothelial cells, afferent arterioles, collecting ducts of kidney, and glomerular mesangial cells [90]. The effect of Ang 1–7 on vascular beds is due to local synthesis of Ang 1–7 especially by endothelium, which mediates an array of vasodilators including EDHF and NO. However, in a patient study with heart failure treated with ACE inhibitor, local infusion of Ang 1–7 failed to induce a local vasodilation [91]. Indeed, losartan effects on decreasing blood pressure are thought to be mediated, partly, via Ang 1–7, since plasma level of Ang 1–7 was found to be increased during ACEI or ARB treatment [13]. The regulation of ACE2 and MasR, at least in hypertensive heart and kidney, was shown to be insensitive to Ang II or aldosterone signaling [92]. These data suggest RAAS-independent ACE2 and MasR regulation. Nevertheless, ACE2/Ang 1–7/MasR signaling has the potential to be considered a novel therapeutic approach to counterbalance ACE/Ang II/AT1R axis as a novel approach targeting RAAS [87].

### 4.5. Angiotensin Receptors Blockers (ARBs)

Blockade of RAAS with ARBs has been shown to reduce inflammation and to improve endothelial function.* In vitro* and* in vivo* studies demonstrate that anti-inflammatory effect of ARB candesartan is through the suppression of the inflammatory toll-like receptors 2 and 4 (TLR2 and TLR4) [17]. Indeed, TLRs have been implicated in development and progression of cardiovascular disease. In animal models of hypertension, TLR4 contributes to blood pressure regulation and small resistance arteries vasoconstriction [93]. In hypertensive patients, ARB irbesartan has been shown to improve endothelial function and vascular reactivity and to reduce the levels of CRP, ICAM-1, IL-6, and oxidative stress marker 8-isoprostane [94]. It is well known that oxidative stress plays an important role in mediating endothelium dysfunction. Use of valsartan has been shown to prevent the formation of reactive oxygen species (ROS) and to suppress the activity of NF-*κ*B, a transcription factor that regulates the expression of inflammatory cytokines and cell adhesion molecules, all of which contribute to development of vascular inflammation and of vascular events [95].* In vitro* and* in vivo* studies have shown that ARB olmesartan inhibits Ang II-induced aortic vascular smooth muscle cells migration and therefore prevents vascular remodeling [96]. Use of ARBs, in patients with type 2 diabetes mellitus or with stable coronary artery disease, increases the number of cardioprotective and endothelial progenitor cells [97, 98]. ARBs exert beneficial effects for the treatment of coronary disease and atherosclerosis. Losartan treatment improves flow-mediated coronary artery disease in patients with atherosclerosis and endothelial function via NO bioavailability.

### 4.6. Mineralocorticoid Receptor Antagonists (MRA)

Dysregulated mineralocorticoid system signaling influences hypertension, atherosclerosis, and cardiac failure independent of renal MR actions on blood pressure [99]. Aberrantly activated MR negatively modulates endothelium function in patients with cardiovascular risk factors and disease. Blocking aldosterone effects at the level of MR with the currently available antagonists, eplerenone and spironolactone, have been shown to be effective treatment options for hypertension and heart failure [100]. The effect of aldosterone on cardiovascular system is mediated via MR present in vascular smooth muscle cells, endothelial cells, and cardiomyocytes [101]. Studies have shown that activation of MR induces a plethora of signaling events in cardiovascular system including oxidative stress [102], inhibits vascular relaxation, and induces vascular inflammation, fibrosis, and remodeling [62]. Aldosterone-activated MR in human endothelial cells (EC) induces the expression of inflammatory factor ICAM-1 and leukocytes-EC adhesion, and blocking MR with spironolactone inhibits aldosterone-mediated effects on EC [102]. Aldosterone impairs EPC differentiation, migration, and proliferation, whereas pharmacological inhibition or genetic manipulation of aldosterone rescues EPC functions [103, 104]. Inhibition of MR with spironolactone improves endothelium-dependent vasodilatation via inhibition of NAD(P)H oxidase pathway [104]. Moreover, blocking aldosterone signaling improves heart muscles proliferation and arterial wall remodeling, endothelial function, and NO synthesis [60]. Many clinical studies have shown that pharmacological inhibition of MR decreases the incidence of heart attack, stroke, and mortality in addition to lowering blood pressure [101]. Most recent studies also show that additional benefits from blockade of aldosterone signaling particularly decreased inflammation, reduced cardiovascular remodeling, and reduced atherosclerosis [60]. However, use of MRA has been shown to indeed induce a countereffect such as an increase in aldosterone level, therefore potentiating MR-independent effects of aldosterone, particularly nongenomic effects such as stimulating contractility of CV system [105].

#### 4.6.1. Development of New Mineralocorticoid Receptor Antagonists

Current research is underway to develop new MRA due to limited number of clinically approved MRA, spironolactone and eplerenone in USA and canrenone in Europe. MR structure-based drug design studies have identified several compounds; however, only a limited number of new MRA candidates have been advanced to clinical studies. One such new MRA, PF-3882845, significantly attenuated blood pressure, reduced urinary albumin, and protected kidney in Dahl-salt induced hypertension animal model. These results advanced this new MRA to ongoing clinical studies [106]. More recent MR antagonists such as dihydrofuran-1-one and dihydropyrrol-2-one show very promising MR binding selectivity in* in vitro* studies [107]. However, other recent selective nonsteroidal molecule, BR-4628, has been developed and displays high potency and selectivity for MR in both,* in vivo* and* in vitro*, studies [108]. In DOCA-salt hypertensive rats, BR-4628 treatment significantly decreased serum inflammation marker MCP-1, IL-1beta, CXCL-1, and kidney damage markers MCP-1, tenascin-1, and osteopontin. Moreover, BR-4628 treatment decreased DOCA-salt induced oxidative stress and DNA damage. However, BR-4628 treatment produced a modest decrease in systolic blood pressure in DOCA-salt group, whereas spironolactone significantly decreased systolic blood pressure compared to DOCA-salt group. These findings suggest tissue activation of MR and BR-4628 antagonism in tissue, rather than systemic effect such as decreasing blood pressure [109]. More studies are necessary to determine BR-4628 efficacy in reducing blood pressure.

### 4.7. Aldosterone Synthase Inhibitors

Decreasing aldosterone synthesis at its enzyme step, aldosterone synthase (AS), CYP11B2, is the novel alternative approach to MRA to limit aldosterone effects [105]. Aldosterone synthase inhibitors (ASI) represent the latest therapeutic strategy to decrease aldosterone production [110]. Fadrozole 286A (FAD 286A) has been shown to dose-dependently inhibit Ang II-induced aldosterone synthesis in human adrenocortical carcinoma cells, whereas in animal models it decreased plasma renin activity, cardiac hypertrophy, and cardiac remodeling [111–113]. Based on FAD 286A, a new AS inhibitor, LCI699, has been developed and is the first generation ASI that has been tested in clinical trials. In clinical trials, administration of LCI699 has been shown to reduce plasma and urine aldosterone level in participants with sodium-depleted levels, to lower blood pressure in patients with essential hypertension and to balance hypokalemia in patients with aldosteronism [110, 114, 115]. Moreover, LCI699 has been recently shown to modestly reduce blood pressure (BP) in patients with resistant hypertension [116]. However, in a study by Karns et al. [116], pharmacological effect of LCI699 was tested on primary aldosteronism, resistant and uncontrolled hypertension, and essential hypertension [106]. In this study, the first generation of ASI, LCI699, has been found to exhibit some limitations, that is, to interfere with major endocrine feedback loops: the RAAS and hypothalamic-pituitary-adrenal (HPA). LCI699 potentiated a higher than accepted physiological level of 11-deoxycorticosterone and adrenocorticotropic hormone (ACTH). The increased levels of 11-deoxycorticosterone and ACTH are components of two different endocrine feedback loops which indeed converge at one point: a potent mineralocorticoid receptor, data which supports a modest LCI699 effect on lowering blood pressure. This data also suggests the need for more* in vivo* studies to fully understand the physiological effect of LCI699.

## 5. RAAS and Vascular Remodeling

Ang II also induces vascular remodeling, thrombosis, and plaques rupture [117, 118]. Ang II mediated vascular remodeling is via the expression of autocrine growth factors basic fibroblast growth factor (bFBS), transforming growth factor-*β*1 (TGF*β*1), and insulin growth factor (IGF) [119]. Vascular remodeling mediated by Ang II is due to increased vascular cell migration and modification of extracellular matrix composition [96, 120]. Changes in the structure and function of blood vessels especially in small resistance blood vessels potentiate the complications of hypertension. Moreover, remodeling of small blood vessels occurs before left ventricular hypertrophy and carotid artery intima-media thickening or increases microalbuminuria levels. Indeed, a smaller lumen and external diameter of small resistance arteries is seen in patients with hypertension [121]. Changes in the function of small arteries are associated with decreased levels of vasodilators and increased sensitivity to Ang II and related signal transduction pathways.

In addition to its role in hypertension, the renin-angiotensin-aldosterone system also plays an important role in mediating vascular remodeling in neointimal hyperplasia after angioplasty and atherosclerosis [122–124]. Ang II is also a growth factor that regulates cell proliferation and differentiation, hypertrophy, and apoptosis. In vascular remodeling, Ang II-induced remodeling effects are due to vascular smooth muscle cells proliferation and hypertrophy. The Ang II growth effects, proliferation versus hypertrophy, are dependent on cell type and cell-cycle regulated genes. For example, Ang II exerts hypertrophic effect on cardiomyocytes via TGF*β*1 mediated signaling, and blockade of TGF*β*1 receptor abrogates Ang II-mediated cardiomyocytes hypertrophy [125]. In myocardial infarction model (MI), ARB, telmisartan, inhibits cardiac remodeling by reducing cardiomyocytes hypertrophy and fibrosis via an anti-inflammatory effect and activation of peroxisome proliferators-activated receptor gamma (PPAR*γ*) [126]. The role of systemic and local renin-angiotensin system in vascular remodeling diseases such as atherosclerosis and neointima hyperplasia after angioplasty is well established [127]. Ang II/AT1R activation within vascular tissue leads to accumulation of inflammatory cells, fibrosis, and migration of vascular smooth muscle cells [128]. Blocking of Ang II signaling via ACEIs or ARBs has been shown to inhibit Ang II-mediated endothelial dysfunction and atherosclerosis [129], and ARBs efficiently inhibited vascular remodeling and neointimal hyperplasia after vascular injury [130, 131]. Indeed, ARB telmisartan has been shown to suppress neointima hyperplasia after heart transplant in a mouse model, suggesting that telmisartan might provide positive effects in preventing graft rejection [132]. ARB losartan has been shown to decrease intima : media ratio of carotid artery or in resistance arteries in hypertensive patients and to prevent the production of TGF*β*, a known mediator of Ang II [133, 134]. Aldosterone plays a key role in cardiac fibrosis and remodeling via direct effects on collagen synthesis and deposition in the cardiac interstitium. Use of spironolactone in rat model of myocardial fibrosis prevents myocardial fibrosis [135]. Therefore, there is increasing evidence indicating that pharmacologically blocking RAAS signaling positively regulates vascular inflammation and remodeling and cardiovascular physiology (for summary of above results, see Table 1).

## 6. Perspective on RAAS Inhibition

Although RAAS system has been studied for more than a century, new experimental and clinical evidences suggest that the physiology of RAAS is complex and multifactorial, and new or on-going research will provide a better understanding on RAAS physiology. For example, the current understanding about interactions between Ang II and aldosterone to regulate deleterious cellular processes indicates the intricacy of RAAS signaling and provides the basis for blocking RAAS at multiple levels. Although new RAAS targets and inhibitors are being developed, more studies are needed to identify the molecular mechanisms and physiological interactions. Moreover, identifying novel targets/effectors of the RAAS system perhaps will provide the basis for the development of novel therapeutic strategies aimed at preventing vascular inflammation and remodeling and therefore improving the outcome of cardiovascular disease.

## Figures and Tables

**Table 1:** Pharmacological effects of RAAS inhibitors on cardiovascular function.

DRI	ACEI	ARB	MRB	Ang 1–7	ASI	(P)RRI
↓ Cardiac hypertrophy	↑ Endothelial function	↑ Endothelial function	↓ Heart attack	Vasodilation	↓ Blood pressure	
↓ Blood pressure	↑ NO	↓ Inflammation	↓ Blood pressure	↓ Proliferation		
↑ Endothelial Function	• Bradikynin	↓ Oxidative stress	↓ Inflammation	↓ Cardiac fibrosis	↓ Cardiac remodeling	↓ Cardiac fibrosis
↓ Monocytes Adhesion	↓ Vascular remodeling	↓ Cardiac fibrosis	↓ Atherosclerosis	↓ Hypertrophy	↓ Cardiac hyperthrophy	↓ Retinal inflammation
↓ Inflammation	• EDHF	↓ Vascular remodeling		↓ Vascular remodeling		
↓ Vascular intima hypertrophy						
↓ Oxidative stress						

ACEI: angiotensin-converting enzyme inhibitors; ARB: type 1 Ang II receptor blockers; MRB: mineralcorticoid receptor blockers; DRI: direct rennin inhibitors; NO: nitric oxide; EDHF: endothelium derived hyperpolarizing factor. Thin arrow: increased or decreased; bullet: maintenance of the level.

## Abbreviations

AS: Aldosterone synthase
ASI: Aldosterone synthase inhibitors
Ang: Angiotensin
ACE: Angiotensin converting enzyme
ARB: Angiotensin receptor blockers
CRP: C-reactive protein
CVD: Cardiovascular disease
DRI: Direct rennin inhibitors
EPC: Endothelial progenitor cells
ERK: Extracellular receptor kinase
EGFR: Epidermal growth factor receptor
eNOS: Endothelial nitric oxide synthase
EDHF: Endothelium-derived hyperpolarizing factor
ET-1: Endothelin 1
IL-1*β*: Interleukin 1 beta
IL-6: Interleukin 6
ICAM-1: Intracellular cell adhesion molecule 1
IGF: Insulin growth factor
MCP-1: Monocytes chemoattractant protein 1
MIP-1: Macrophages inflammatory protein 1
MRA: Mineralocorticoid receptor antagonist
NF-*κ*B: Nuclear factor kappa B
NO: Nitric oxide
PPAR*γ*: Peroxisome proliferators-activated receptor gamma
RAAS: Renin-angiotensin-aldosterone system
TGF*β*: Transforming growth factor beta
TNF*α*: Tumor necrosis factor alpha
VCAM-1: Vascular cell adhesion molecule 1.
